# Relationships between Fitness Status and Match Running Performance in Adult Women Soccer Players: A Cohort Study

**DOI:** 10.3390/medicina57060617

**Published:** 2021-06-13

**Authors:** Lillian Gonçalves, Filipe Manuel Clemente, Joel Ignacio Barrera, Hugo Sarmento, Francisco Tomás González-Fernández, Luiz H. Palucci Vieira, António José Figueiredo, Cain C. T. Clark, J. M. Cancela Carral

**Affiliations:** 1 Faculty of Educational Sciences and Sports Sciences, University of Vigo, 36005 Pontevedra, Spain; chemacc@uvigo.es; 2 Escola Superior Desporto e Lazer, Instituto Politécnico de Viana do Castelo, Rua Escola Industrial e Comercial de Nun’Álvares, 4900-347 Viana do Castelo, Portugal; filipe.clemente5@gmail.com; 3 Instituto de Telecomunicações, Delegação da Covilhã, 1049-001 Lisboa, Portugal; 4 Research Unit for Sport and Physical Activity, Faculty of Sport Sciences and Physical Education, University of Coimbra, 3004-531 Coimbra, Portugal; jibarrera@outlook.es (J.I.B.); hg.sarmento@gmail.com (H.S.); afigueiredo@fcdef.uc.pt (A.J.F.); 5 Department of Physical Activity and Sport Sciences, Pontifical University of Comillas (Centro de Estudios Superiores Alberta Giménez), 07013 Palma, Spain; francis.gonzalez.fernandez@gmail.com; 6 MOVI-LAB Human Movement Research Laboratory, School of Sciences, Graduate Program in Movement Sciences, Physical Education Department, UNESP São Paulo State University, Bauru 01140-070, Brazil; luiz.palucci@unesp.br; 7 Centre for Intelligent Healthcare, Coventry University, Priory St, Coventry CV1 5FB, UK; cain.clark@coventry.ac.uk

**Keywords:** football, athletic performance, match analysis, sports training, GPS, high-intensity running

## Abstract

*Background and Objectives:* The aim of this study was twofold: (i) to analyze the relationships between fitness status (repeated-sprint ability (RSA), aerobic performance, vertical height jump, and hip adductor and abductor strength) and match running performance in adult women soccer players and (ii) to explain variations in standardized total distance, HSR, and sprinting distances based on players’ fitness status. *Materials and Methods*: The study followed a cohort design. Twenty-two Portuguese women soccer players competing at the first-league level were monitored for 22 weeks. These players were tested three times during the cohort period. The measured parameters included isometric strength (hip adductor and abductor), vertical jump (squat and countermovement jump), linear sprint (10 and 30 m), change-of-direction (COD), repeated sprints (6 × 35 m), and intermittent endurance (Yo-Yo intermittent recovery test level 1). Data were also collected for several match running performance indicators (total distance covered and distance at different speed zones, accelerations/decelerations, maximum sprinting speed, and number of sprints) in 10 matches during the cohort. *Results*: Maximal linear sprint bouts presented large to very large correlations with explosive match-play actions (accelerations, decelerations, and sprint occurrences; *r* = −0.80 to −0.61). In addition, jump modalities and COD ability significantly predicted, respectively, in-game high-intensity accelerations (*r* = 0.69 to 0.75; R^2^ = 25%) and decelerations (*r* = −0.78 to −0.50; R^2^ = 23–24%). Furthermore, COD had significant explanatory power related to match running performance variance regardless of whether the testing and match performance outcomes were computed a few or several days apart. *Conclusion*: The present investigation can help conditioning professionals working with senior women soccer players to prescribe effective fitness tests to improve their forecasts of locomotor performance.

## 1. Introduction

Soccer matches represent a well-known intermittent mode of exercise in which short periods of intense efforts are interspaced by periods of low-to-moderate intensity [1,2]. Thus, players must maintain a desired level of running intensity and recover rapidly to perform to the best of their ability [3,4]. The literature has demonstrated that women soccer players may cover 9 to 11 km per match while spending 99 ± 8.3 m∙min^−1^ performing low-speed running and 9.7 ± 3.7 m∙min^−1^ performing high-speed running [2,5,6,7,8].

In female soccer, sprinting is considered a high-intensity effort [9], and high-speed activity is considered an essential component of matches. Usually, such efforts occur during decisive moments in a match [7], though they represent only 8% to 12% of the total distance covered in a typical match [10]. Additionally, female players were found to perform between 70 and 190 high-intensity runs (>19.8 km∙h^−1^) during a match [5,10,11], covering between 210 and 520 m [6,7,12,13].

To sustain such efforts, female soccer players should present well-developed fitness statuses that allow them to meet the various demands of a match. Regarding sprinting performance, typical fitness status values observed in women soccer players suggest that they can cover 10 m in 2.31 ± 0.21 s and 25 m in 4.52 ± 0.20 s [14,15,16,17]. For another determinant variable (i.e., lower limb power), typical values exhibited by women soccer players are 30.1 ± 3.7 cm in the squat jump and 31.6 ± 4.0 cm in the countermovement jump [18]. Both sprinting and lower-limb power are neuromuscular determinants of soccer performance. However, the sport overwhelmingly involves running at low-to-moderate intensities—thus, good cardiorespiratory performance is crucial.

Female players usually present maximal oxygen uptake values between 49.4 and 56.7 mL/kg/min [2,17]. Based on one of the most common field-based tests used in soccer (namely, the Yo-Yo intermittent recovery test level 1), elite women soccer players can cover 1224 ± 255 m during the test, while players from lower divisions cover 826 ± 160 m [2,17].

Since high-intensity runs and sprinting tend to decrease at the end of the match, they could be associated with fatigue [19,20,21,22]. Therefore, sustaining good aerobic levels can help players avoid the effects of fatigue when performing power-related actions. Naturally, a player’s performance will be affected by multiple factors, such as their position [23,24]. For instance, research indicates that central defenders perform fewer high-intensity runs than other players [23,24]. 

Fitness status can support match running performance—however, the strength of this relationship differs depending on the type of demand imposed on the player and the physical quality. For example, repeated sprint ability seems to be significantly correlated with total and high-intensity distances covered in matches [23,24,25,26,27,28,29,30,31,32,33,34,35,36,37,38]. Total distance also presented large correlations with high-intensity running activities and aerobic performance in field-based tests performed by male and female youth soccer players [29,30,31,32,33,34,35,36,37,38,39,40,41,42]. 

However, very few studies have tested the relationships between fitness status and match running performance among female soccer players. Nevertheless, it is pertinent to consider which kind of fitness status best relates to specific efforts in matches since match running performance is a determinant of a player’s ability to sustain a high performance level. Understanding this matter will help to emphasize and specify the training process. However, fitness status changes over time. As such, analyzing the relationships between match running performance and fitness status in different moments throughout a season can help to explore whether these relationships are influenced by time.

Following the above discussion, the present study aims to (i) analyze the relationships between fitness status (repeated sprint ability (RSA), aerobic performance, vertical jump height, and anthropometry) and match running performance and (ii) run a regression analysis to explain variations in total distance, high-speed running (HSR), and sprinting distance. The hypothesis of the study is that match running variables are explained by the fitness status of the players.

## 2. Materials and Methods

### 2.1. Experimental Approach to the Study

**Figure 1:**
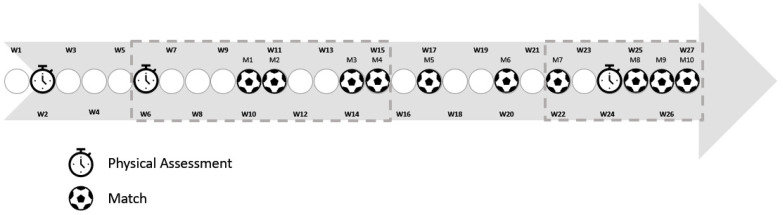
Timeline of the study.

This 22-week study followed an observational analytic cohort design. Players were assessed three times during the cohort (Figure 1). The first and second assessments were separated by four weeks, whereas the second and third assessments were separated by 18 weeks. The intervals were varied to determine the relationship between the physical capacities assessed with the match running and variations observed in total distance, HSR, and sprinting distance during matches.

Three participants were excluded from the analysis, and 22 participants remained. Assessment 2 was correlated with matches 1 to 4 (weeks 6 to 15), while assessment 3 was correlated with matches 7 to 10 (weeks 22 to 27). A correlation analysis was conducted between fitness variables and match running performance for each period of assessment. Additionally, multi-linear regression analysis was carried out considering the match running performance variables to determine how each of the three variables of interest influenced running performance.

### 2.2. Participants

Twenty-two women soccer players from a team participating in the first Portuguese league were observed (Table 1). The participants presented a mean age of 24.77 ± 6.49 years old and a height of 162.51 ± 7.08 cm. In the first assessment mean weight was 59.06 ± 9.50 kg. In the second assessment mean weight was 59.01 ± 9.30 kg and body mass 61.62 ± 9.50 kg. The sample included three goalkeepers, four external defenders, four central defenders, six midfielders, and five attackers. During the season, players participated in four training sessions per week and official matches on weekends.

The eligibility criteria that players had to meet to be included in the final sample were as follows: (i) completion of all three assessments; (ii) participation in at least 85% of training sessions, (iii) not being out of action for treatment for more than four weeks, and (iv) at least five years of experience.

Before the study began, all players were informed of the study’s design and procedures. Afterward, each player signed an informed consent form. The study was approved by the local university (code: CTC-ESDL-CE001-2021; date: 18 March 2021) and followed the ethical standards as per the Declaration of Helsinki for studies involving humans.

### 2.3. Measures

#### 2.3.1. Physical Fitness Assessment

Between August and January, three fitness assessments with similar demands occurred in three microcycles. For each assessment period (week), three days were dedicated to run the tests, interspaced by 24 h between them. Players had 48 h of rest before the first day of assessments of each week analyzed.

In the first training session of the week, players were tested for anthropometry and hip adductor and abductor strength. In the second training session, vertical jump height, changes of direction, and linear speed were assessed. In the third session, the repeated sprint ability test and the Yo-Yo intermittent recovery test level 1 were applied.

These tests always occurred at the same time (7:30 p.m.) and location. The linear speed, repeated sprint ability, and Yo-Yo intermittent recovery tests were performed on synthetic turf without rain at a mean temperature of 19.5 ± 3.4 °C and a relative humidity of 63 ± 4%. A warm-up was performed before all evaluations. Warm-ups consisted of low and self-paced running, followed by calisthenic exercises in which players performed two sets of 10 repetitions of walking lunges, single-leg deadlifts, and fontal and lateral high knee movements.

##### Anthropometry

Body weight (kg) was measured without shoes with a bioelectrical impedance analysis (BIA) device (Tanita BC-730) to the nearest 0.1 kg. Height (cm) was measured using a stadiometer (Type SECA 225, Hamburg, Germany) to the nearest 0.1 cm.

##### Repeated Sprint Ability

The running anaerobic sprint test (RAST) test was applied to test players’ repeated sprint abilities. This test consisted of six runs of 35 linear meters (each interspaced by 10 s of rest), with no COD required [43]. The time (sec) of each effort was recorded using a photocell timing gate (Photocells, Brower Timing System, UT, USA), with one device positioned at the starting line and the other positioned at the finish line. The device had a resolution of one-thousandth of a second. The minimum and maximum peak power and the fatigue index were determined using the following equation [43]: $\mathrm{Power}\;=\frac{\mathrm{Weight}\;\times {\;\mathrm{Distance}}^{2}}{{\mathrm{Time}}^{3}}$ and $\mathrm{Fatigue}=\frac{\max_{\mathrm{power}}-{\;\min}_{\mathrm{power}}\;}{\mathrm{Sum}\;\mathrm{of}\;6\;\mathrm{sprints}\;\left(s\right)}$.

##### Linear Sprinting

Players’ 10- and 30-m linear sprint abilities were tested using photocell timing gates (Photocells, Brower Timing System, UT, USA) positioned at the start and finish lines. Participants began the test positioned 0.5 m behind the starting line in a two-point split stance. As with the repeated sprint test, the device used to measure the players’ performance had a resolution of one-thousandth of a second. Each player’s best result obtained from three separate trials was recorded as their sprint time. 

##### Change-of-Direction

The zig-zag 20 m [40] test was used to assess COD. This test consists of four 5 m each set out at 100°. Times were once again recorded using photocells timing gates (Photocells, Brower Timing System, UT, USA) with a resolution of one-thousandth of a second. The typical error of the Photocells was between 0.04 and 0.06 s, while the smallest worthwhile change was between 0.11 and 0.17 s [41]. Subjects performed three trials, resting for at least three minutes between trials. The best time (lowest time in seconds) of the three trials was used for the analysis.

##### Squat and Countermovement Jump

Squat and countermovement jump heights were assessed, with the highest jumps (cm) recorded and used in the analysis. Both jumps were tested with an optical measurement system consisting of a transmitting and receiving bar (Optojump, Microgate, Bolzano, Italia). 

Each participant started the squat jump test in a squat position (although self-selected, the recommendation was to stay approximately at 90° relative knee joint angle) with their hands on their waist. After spending three seconds in the squat position, the participant jumped by extending their legs and then landed in the same place. Each participant performed three trials, with 30 s of rest provided between jumps.

Each participant started the countermovement jump test from a standing position, with their hands on their waist. After spending three seconds in the standing position, the participant flexed their legs and then immediately extended them while jumping. Each participant performed three trials, with 30 s rest provided between jumps.

##### Yo-Yo Intermittent Recovery Test—Level 1

For the Yo-Yo Intermittent Recovery test, participants were to run 20 m from one mark to another and then return to the starting mark. After every 40 m covered, a 10-s recovery period is provided, during which time participants jog between two marks that are five meters apart (an audio beep is utilized to control participants’ speed). The speed starts at 10 km/h, increasing progressively thereafter. The test ends when the athlete achieves voluntary exhaustion or does not reach one of the 20-m marks before or at the same time as the beep. At the end of the test, the number of completed levels and shuttles, as well as the total distance covered, were recorded. The total distance (meters) was recorded. 

##### Hip Adductor and Abductor Strength

A dynamometer (Smart Groin Trainer, Neuro excellence, Portugal) was positioned on the thigh area of participants, who were asked to squeeze the tool for 20 s. Three trials were performed, with 10 s of rest between trials. The strength of the hip adductor and abductor was measured in kilograms. The highest value was used in the analysis. 

#### 2.3.2. Match Running Performance

During the match, participants used a Global Position System (GPS) (SPI HPU, GPSports, Canberra, Australia). This device has a frequency of 15 Hz and accelerometer of 100 Hz, 16 G Tri-axis, and a magnetometer of 50 Hz. Participants were asked to use a tight-fitting vest during the match and the device was placed between the left and right scapula. The GPS device collected the speed (km∙h^−1^), the maximal speed (km∙h^−1^), the number of sprints, the time of each sprint (sec), and accelerations and decelerations executed during each match observed. Speed achieved during a match was divided into the following 6 zones: zone 1 (0–5.9 km∙h^−1^), zone 2 (6–11.9 km∙h^−1^), zone 3 (12–13.9 km∙h^−1^), zone 4 (14–17.9 km∙h^−1^), zone 5 (18–23.9 km/h), and zone 6 (>24 km∙h^−1^). The acceleration and deceleration were also recorded and split into 3 zones: ace1 (1.0–1.9 m∙s^2^), ace2 (2.0–2.9 m∙s^2^), ace3 (3.0–4.0 m∙s^2^) and des1 (1.0–1.9 m∙s^2^), des2 (2.0–2.9 m∙s^2^), des3 (3.0–4.0 m∙s^2^). The external load collected for analysis were: total distance covered (m), the distance covered (m) in the different speed zones, accelerations (m∙s^2^), decelerations (m∙s^2^), the maximum speed achieved (km/h), and the number of sprints (n).

### 2.4. Statistical Analysis

Descriptive statistics were represented as mean ± SD. Normal distribution and homogeneity was tested with the Kolmogorov-Smirnov test on all data before analysis. A Pearson correlation coefficient *r* was used to examine the relationship between values of fitness assessment (hip strength (ADDs and ABDs); squat and countermovement jump (SJ and CMJ); change-of-direction test (COD in seconds); linear Sprinting (10 m and 30 m in seconds); repeated sprint ability test (P_max_, P_min_ and FI); Yo-Yo intermittent recovery test 1 (YYIR1 distance)) and match running performance (total distance covered (D); speed achieved in zone 1 (Z1), zone 2 (Z2), zone 3 (Z3), zone 4 (Z4), zone 5 (Z5), and zone 6 (Z6); acceleration (ace1, ace2, ace3) and deceleration (des1, des2, des3); maximum speed achieved (MSA); and number of sprint (NS)). To interpret the magnitude of these correlations we adopted the following criteria: *r* ≤ 0.1, trivial; 0.1 < *r* ≤ 0.3, small; 0.3 < *r* ≤ 0.5, moderate; 0.5 < *r* ≤ 0.7, large; 0.7 < *r* ≤ 0.9, very large; and *r* > 0.9, almost perfect [44]. The changes over the assessment were determined using repeated measures ANOVA. Significant main effects were subsequently analyzed using a Bonferroni post hoc test. Effect size is indicated with partial eta squared for Fs. To interpret the magnitude of the eta squared we adopted the following criteria: η^2^ = 0.02, small; η^2^ = 0.06, medium; and η^2^ = 0.14 large. Regression analysis was used to identify which fitness outcomes can better explain match running performance. All variables were examined separately in this regression analysis. The magnitude of R2 was interpreted as follows: >0.02, small; >0.13, medium; >0.23, large. Data were analyzed using Statistica software (version 10.0; Statsoft, Inc., Tulsa, OK, USA). 

## 3. Results

Descriptive statistics were calculated for each variable (see Table 1 and Table 2 for more information). 

A repeated measures ANOVA with participants’ mean hip strength (ADDs and ABD) did not reveal any effect of assessment *F* > 1, in both cases. Another repeated measures ANOVA with participants’ mean squat and countermovement jump (SJ and CMJ) did not reveal an effect of assessment in SJ, *F* (1.12) = 2.42, *p* = 0.11, η^2^ = 0.16. However, data showed a significant effect of assessment in CMJ, *F* (1.12) = 6.13, *p* = 0.01, η^2^ = 0.33. Continuing with the same type of repeated measures ANOVA analysis with participant ’s mean change-of-direction (COD (s), COD (km∙h^−1^), and COD (m∙s^−1^)) did not reveal any effect of assessment *F* > 1. In the same line, another ANOVA analysis with participants mean linear sprinting (10 m (s), 10 m (km∙h^−1^), 10 (m∙s^−1^), 30 m (s), 30 m (km∙h^−1^), 30 (m∙s^−1^)) did not reveal any effect of assessment *F* > 1. A repeated measures ANOVA with participants’ mean repeated sprint ability test (P_max_ (s), P_min_ (s), P_average_ (s) and FI (%)) revealed an effect of assessment for P_max_ (s), P_min_ (s), and P_average_ (s), *F* (1.12) = 4.86, *p* = 0.01, η^2^ = 0.28, *F* (1.12) = 8.84, *p* = 0.001, η^2^ = 0.42, and *F* (1.12) = 6.23, *p* = 0.01, η^2^ = 0.34, respectively. Nevertheless, there was no effect of assessment for FI (%), *F* > 1. Particularly remarkable, a repeated measures ANOVA with participants’ mean Yo-Yo intermittent recovery test level 1 (stage (n), YYIR1, distance (m), HR_max_ (bpm), and V02_max_ (mL∙kg^−1^∙min^−1^)) revealed an effect of assessment for stage (n), YYIR1, distance (m), and V02_max_ (mL∙kg^−1^∙min^−1^), *F* (1.8) = 7.40, *p* = 0.001, η^2^ = 0.48, *F* (1.8) = 7.40, *p* = 0.001, η^2^ = 0.48, *F* (1.8) = 7.40, *p* = 0.01, η^2^ = 0.42, respectively. However, HR_max_ (bpm) data did not show any effect of assessment, *F* > 1.

The effect of match running performance tested repeatedly (D, Z1, Z2, Z3, Z4, Z5, Z6, ace1, ace2, ace3, des1, des2, des3, MSA and NS = between match 1 to match 4 (n), match 7 to match 10 (n), match 1 to match 4 (n per min), match 7 to match 10 (n per min)) did not reveal any effect of assessment of any studied variable, *F* > 1, in all cases.

On the basis of data obtained, correlations analysis was performed in order to find the possible association between fitness assessment and match running. First, we performed analysis of assessment 2 and matches 1–4, and second, assessment 3 and matches 7–10. Consequently, the correlation between fitness assessment and match running (assessment 2 and matches 1–4) are summarized in Table 3. No significant correlations were found between fitness assessment and the next variables of match running (D, Z1, Z2, Z3, Z4, Z5, ace1, ace2, des1, des2, MSA, and NS). However, negative correlation was found between 30 m linear sprinting and ace3, *r* = −0.52, *p* = 0.24. Crucially, other negative correlations were found between COD and Z6 and ace3 and des3 (*r* = −0.57, *p* = 0.024; *r* = −0.59, *p* = 0.011; *r* = −0.50, *p* = 0.034, respectively). 

Correlation analysis was performed in order to find possible association between fitness assessment and match running (assessment 3 and matches 7–10). All data are summarized in Table 4. No significant correlations were found between fitness assessment and the next variables of match running (D, Z2, Z3, ace1, and des1). Nevertheless, positive correlation was found between SJ and ace3, des2, des3, and NS (*r* = 0.75, *p* = 0.007; *r* = 0.64, *p* = 0.035; *r* = 0.63, *p* = 0.035, and *r* = 0.70, *p* = 0.016, respectively). Other positives correlations were found between CMJ and Z1, Z4, ace2, ace3, des2, des3, and NS (*r* = 0.61, *p* = 0.048; *r* = 0.63, *p* = 0.040; *r* = 0.64, *p* = 0.036, *r* = 0.69, *p* = 0.019, *r* = 0.67, *p* = 0.022, *r* = 0.62, *p* = 0.039, and *r* = 0.70, *p* = 0.016, respectively). Furthermore, negative correlations were encountered between 10 m and ace2, *r* = −0.61, *p* = 0.047; des2, *r* = −0.61, *p* = 0.050; and NS *r* = −0.75, *p* = 0.008. Negative correlations were encountered between 30 m and Z5, Z6, ace2, ace3, des2, des3, MSA, and NS (*r* = −0.63, *p* = 0.039; *r* = −0.70, *p* = 0.016; *r* = −0.68, *p* = 0.021, *r* = −0.77, *p* = 0.006, *r* = −0.68, *p* = 0.022, *r* = −0.68, *p* = 0.022, *r* = −0.68, *p* = 0.023, and *r* = −0.80, *p* = 0.003, respectively). In the same line, more negative correlations were found between COD and Z4, Z5, Z6, ace2, ace3, des2, des3, and NS (*r* = −0.68, *p* = 0.022; *r* = −0.80, *p* = 0.003; *r* = −0.77, *p* = 0.006, *r* = −0.76, *p* = 0.007, *r* = −0.84, *p* = 0.001, *r* = −0.78, *p* = 0.005, *r* = −0.75, *p* = 0.007, and *r* = −0.74, *p* = 0.010, respectively). In addition, another positive correlation was encountered between FI and MSA, *r* = 0.61, *p* = 0.043.

Lastly, a multilinear regression analysis was performed to verify which variable of fitness assessment (agreement with the correlation analysis) could be used to better explain match running performance (See Table 5. for more information).

## 4. Discussion

The main aim of the current study was to determine the magnitude of relationships between various fitness status measures (strength, power, single/repeated sprinting, and intermittent endurance) and match running performance in adult women soccer players competing at a high level. We also aimed to explain the match running variations based on fitness status. The main findings in the present Portuguese players indicate the following: (1) correlations between fitness and match running performance were dependent on the time frame separating the testing battery and the collection of running performance during actual match-play. (2) With only rare exceptions, isolated strength, intermittent endurance, and repeated sprint ability performance were not associated with, nor did they predict, match running performance. (3) Even considering the fact that tests for separate maximal sprint bouts (10 and 30 m) were largely to very largely associated, they failed to significantly explain the variance of match-play (e.g., explosive) locomotor variables. (4) Jump and COD ability clearly allowed a good (medium-to-large) prediction to be obtained of in-game high-intensity accelerations and decelerations, respectively. Finally, (5) the latter evaluation method was the only fitness indicator that had significant power to predict match running performance independent of the interval between testing and match performance.

Some innovative aspects in the current study should be highlighted. First, while the majority of previous works adopted only correlations as a statistical treatment to evaluate the possible link between fitness status and match running performance in soccer [41,45,46,47,48], a decision was made to move further when providing recommendations mainly based on regression analysis, which reveals the weighted influence of players’ physical capacity on their locomotor outputs during match-play. In addition, various investigations on the subject have tested players at a single time point [36,37,38,40,41,49,50]. Meanwhile, here, two distinct approaches were considered using a cohort design, one testing associations between fitness status determined near the match occurrences and another with a longer interval between them. Most importantly, less than 3% of evidence on the complexity of fitness-match running performance relationships in a soccer context [51] were derived from scientific studies including female players according to knowledge collated in reviews [27,52]. Nonetheless, only intermittent endurance (Yo-Yo IR1/IE2), aerobic fitness (laboratory treadmill tests) [2,53], and Wingate measurements [11] were previously related to match running performance in women’s soccer. Again, this reinforces the originality of data presented in the current work and supports the critical appraisal of the findings’ strengths and weaknesses, which is developed in the following paragraphs.

An important finding of the present investigation is that a fitness testing battery seems to have a relatively short expiration date to help preview match physical performance in female soccer players. Such is indicated by the frequency and strength of correlations between fitness status measures and match running performance, as well as the number of variables involved, which varied in the distinct moments. According to a recent critique piece [53,54,55,56,57], manipulating the interval between players’ evaluations and matches was never previously addressed when the objective was to understand their associations. Here, when looking at fitness assessment 2, we noticed only four moderate-to-large (ranging from −0.59 to −0.50) correlations with matches 1 to 4 (e.g., COD and 30 m sprint tests with in-game very-high intensity accelerations) (Table 3). All these were performed across a 10-week period, where fitness tests and matches were separated by three to nine weeks.

In contrast, more than 30 large to very large correlation coefficients (ranging from −0.84 to 0.75) were found between fitness assessment 3 parameters and running outputs during matches 7–10 (e.g., SJ, CMJ, 10/30 m sprint and COD with in-game sprint occurrences) (Table 4). Such second analysis comprised a 6-week period in total, with an interval between test and match equal to no more than three weeks. Naturally, changes occurring between fitness assessments were not explained in this work, but possibly may be affected by the training process [26,58,59]. Reports have not yet confirmed the same for match running performance, even though these are related to each other crosswise [3,15,57]. However, based on the current observations, the usefulness of some fitness data may become outdated (or at least its relevance might be reduced) after approximately a month in women’s soccer.

The local strength of hip adductor and abductor muscles and intermittent high-intensity or endurance running bouts were not associated, nor were they predictors. However, 10- to 30-m sprint performances were largely to very largely related to match running outputs in the female players of the present study, though their shared variance had no statistical significance.

When comparing such results to those presented in the available literature, discrepancies are identified. For example, this was the case in intermittent endurance capacity, which was previously linked to match running performance in female senior players [3,57] as well as in senior and youth male populations [27]. However, recent studies have demonstrated that running outputs during small-sided games were associated with the outputs obtained in competitions [54] independent of the player’s intermittent endurance profile [55]. Force, maximal velocity, and aerobic and anaerobic resistance are important fitness components arguably contributing to sustaining physical efforts experienced in soccer match-play, thereby representing frequent determinants to winning [56,57].

Notwithstanding, many issues likely compromise the utility of some fitness assessment protocols in the current format. For example, testing maximal sprint ability (single or successive running bouts) using only linear paths is criticized nowadays given the curved trajectory of most explosive in-game actions [58]. The very short intervals often offered between repeated all-out efforts do not match those encountered in actual matches [59]. Occurrences of near-to-maximal displacements can also be very uncommon in elite standards [60]. Hip adduction/abduction strength allows one to discriminate between distinct performance levels [61], yet the effective contribution of hip muscle strength to running kinematics is low [62,63]. To summarize, such points of view are in alignment with our results. The external validity of some popular fitness status markers in women’s soccer is not always supported, and its indiscriminate use needs to be re-thought.

Conditioning professionals need to be aware of assessment tools that can remain consistent over time when classifying players based on their fitness status, as well as the potential implications in terms of match performance. In this sense, although the construct validity of the various methods tested here has been challenged, jump and COD ability provided reasonable predictions (R^2^ ranging from 23 to 25%, respectively) (Table 5) for in-game high-intensity accelerations and decelerations. Furthermore, COD predicted match running performance regardless time between testing and match observations. In other words, regression models with inputs being COD data remained significant from assessment 2/matches 1–4 until assessment 3/matches 7–10.

Studies that have aimed to extract the most relevant game indicators in soccer suggest that accelerations and decelerations are among the main components of athletes’ external loads [64]. Interestingly, decelerations are more frequent than accelerations in soccer match-play [65]. In addition to being paramount to COD performance, skilled decelerations are also fundamental to a range of match events (e.g., rapid changes in speed, cutting maneuvers, and regaining ball possession) [66]. Therefore, change-of-direction seems to be a sensitive indicator of fitness status in female soccer players, as it may provide meaningful information about the next match profile, in particular the players’ deceleration performance.

Aside from the novelty of the current investigation, a number of limitations are recognized and need to be accounted for in future work, as well as when making interpretations and generalizations based on the present evidence. For one, female players were grouped regardless of the general exertion of their positional role during matches. Studies in male soccer players have shown that fitness status and match running performance relationships can be position dependent [25,26,53]. Another limitation is that the recommended sample size (*N* = 80 players) as per Gregson et al. [67] was not met in the present investigation, though this has frequently been the case in similar research. It is possible given the practical difficulty of involving multiple clubs that the pertinence of large/potentially heterogeneous datasets in solving this problem also lacks consensus. In addition, none of the conducted tests required players to rely on technical-tactical performance. Instead, they evaluated physical capacity markers. Adopting protocols that more closely mimic game demands could enhance the ecological validity and, in turn, the predictive ability of a testing battery in informing to some extent and advancing physical performance during matches [54]. Finally, the games involved different opposition over the course of the study, which may have impacted the running demands completed in matches.

## 5. Conclusions

Including a change-of-direction ability test seems pertinent when assessing women soccer players, as it may partly predict match-play running performance regardless of whether the time separating the assessment and competition is shorter (testing immediately prior to/after competing) or longer (test-match moments interspaced by at least three weeks). It is something that provides preliminary evidence about the construct validity of COD testing and its likely robustness regarding common time-related changes in match running performance.

We also demonstrated that the further apart a fitness testing battery is carried out in relation to actual matches, the lower its value in predicting in-game running outputs. Finally, caution is required concerning conditioning professionals’ extensive use of common testing procedures, such as isolated maximal sprints, intermittent high-intensity actions, or endurance bouts, as evidence in the present study revealed that these do not always provide useful information for forecasting inter-individual variations in match-play locomotor performance.

## Figures and Tables

**Table 1:** Physical fitness assessment (mean ± SD).

Measure	Women Soccer Players (*n* = 22)		
	Assessment 1	Assessment 2	Assessment 3
**Hip strength**			
ADDs (kg)	-	34.66 ± 7.81	35.81 ± 7.22
ABDs (kg)	-	33.48 ± 5.87	34.40 ± 6.03
**Squat and countermovement jump**			
SJ (cm)	25.33 ± 2.98	26.24 ± 3.09	23.85 ± 4.29
CMJ (cm)	27.26 ± 2.97	27.40 ± 3.51	24.17 ± 4.16
**Change-of-direction test**			
COD (s)	5.73 ± 0.19	5.75 ± 0.18	5.80 ± 0.22
COD (km∙h^−1^)	12.60 ± 0.40	12.53 ± 0.39	12.42 ± 0.46
COD (m∙s^−1^)	3.50 ± 0.11	3.48 ± 0.10	3.45 ± 0.12
**Linear Sprinting**			
10-m (s)	1.87 ± 0.08	1.90 ± 0.10	1.88 ± 0.10
10-m (km∙h^−1^)	19.29 ± 0.84	18.98 ± 0.98	19.13 ± 0.99
10-m (m∙s^−1^)	5.36 ± 0.23	5.27 ± 0.27	5.31 ± 0.27
30-m (s)	4.79 ± 0.22	4.77 ± 0.21	4.75 ± 0.23
30-m (km∙h^−1^)	22.57 ± 1.05	22.64 ± 0.99	22.75 ± 1.05
30-m (m∙s^−1^)	6.27 ± 0.29	6.29 ± 0.27	6.31 ± 0.29
**Repeated sprint ability test (RSA test)**			
P_max_ (s)	380.81 ± 68.38	401.77 ± 74.47	448.63 ± 64.99
P_min_ (s)	240.44 ± 46.87	267.15 ± 46.29	295.53 ± 34.68
P_average_ (s)	305.21 ± 48.93	321.83 ± 50.53	355.23 ± 39.31
FI (%)	4.61 ± 1.85	4.41 ± 1.65	5.02 ± 1.75
**Yo-Yo intermitteng recovery test- Level 1**			
Stage (n)	14.62 ± 0.65	14.94 ± 0.77	15.15 ± 0.73
YYIR1, Distance (m)	677.78 ± 251.74	788.00 ± 219.89	682.66 ± 397.89
HR_max_ (bpm)	197.58 ± 5.33	197.50 ± 5.33	197.04 ± 5.25
VO2_max_ (mL∙kg^−1^∙min^−1^)	41.79 ± 2.11	43.02 ± 1.85	43.56 ± 1.73

Note: VO2_max_ was estimated by the next equation: Yo-Yo IR1 test: VO2_max_ (mL/min/kg) = IR1 distance (m) × 0.0084 + 36.4 (Bangsbo. 2008); ADD: adductor strength; ABD: abductor strength; SJ: squat jump; CMJD: countermovement jump; COD: change-of-direction; P_max_: maximum power at repeated-sprint test; P_min_: minimum power at repeated-sprint test; P_average_: average power at repeated-sprint test; FI: fatigue index at repeated-sprint test; YYIR1: intermittent recovery test level 1; HR_max_: maximal heart rate; VO2_max_: maximal oxygen uptake.

**Table 2:** Descriptive table of match running performance (mean ± SD).

Measure	Match 1 to Match 4 (*n*)	Match 7 to Match 10 (*n*)	Match 1 to Match 4 (*n* per min)	Match 7 to Match 10 (*n* per min)
D (m)	8091.47 ± 391.16	8383.01 ± 622.60	101.46 ± 9.21	99.00 ± 16.09
Z1 (m)	3143.72 ± 176.67	3283.83 ± 268.36	39.12 ± 3.83	39.07 ± 7.11
Z2 (m)	3189.10 ± 312.05	3183.08 ± 269.28	40.13 ± 4.86	37.37 ± 5.63
Z3 (m)	740.84 ± 51.08	758.21 ± 93.40	9.35 ± 1.02	8.90 ± 1.52
Z4 (m)	721.66 ± 40.98	792.56 ± 92.23	9.14 ± 0.85	9.36 ± 1.72
Z5 (m)	273.78 ± 31.33	339.08 ± 51.93	3.44 ± 0.24	4.00 ± 0.90
Z6 (m)	19.64 ± 8.16	23.78 ± 9.91	0.24 ± 0.10	0.27 ± 0.12
ace1 (*n*)	172.31 ± 10.76	175.53 ± 21.11	2.16 ± 0.20	2.06 ± 0.35
ace2 (*n*)	40.68 ± 4.63	41.48 ± 6.56	0.52 ± 0.07	0.49 ± 0.11
ace3 (*n*)	2.69 ± 0.29	2.50 ± 0.47	0.03 ± 0.00	0.03 ± 0.01
des1 (*n*)	146.21 ± 9.68	146.37 ± 16.34	1.84 ± 0.17	1.71 ± 0.25
des2 (*n*)	44.24. ± 4.75	43.91 ± 7.91	0.56 ± 0.08	0.52 ± 0.13
des3 (*n*)	14.40 ± 1.80	15.36 ± 3.03	0.18 ± 0.12	0.18 ± 0.05
MSA (km∙h^−1^)	24.00 ± 0.38	24.49 ± 1.35	24.00 ± 0.38	24.49 ± 1.35
NS (*n*)	14.74 ± 8.99	14.62 ± 0.65	0.19 ± 0.12	0.20 ± 0.04

Note: *n* per minute was calculated considering the time in match; NS: number of sprints; MSA: maximum speed achieved; zone 1 (Z1), zone 2 (Z2), zone 3 (Z3), zone 4 (Z4), zone 5 (Z5), and zone 6 (Z6); acceleration (ace1, ace2, ace3) and deceleration (des1, des2, des3); D: distance covered.

**Table 3:** Correlations between fitness assessment and match running (assessment 2 and matches 1–4).

Measure	D	Z1	Z2	Z3	Z4	Z5	Z6	ace1	ace2	ace3	Des1	Des2	Des3	MSA	NS
ADD (kg)	0.19	0.20	0.14	0.15	0.26	0.31	0.19	0.18	0.36	0.17	0.20	0.20	0.23	0.24	0.33
ABD (kg)	0.26	0.37	0.21	0.07	0.15	0.14	0.07	0.17	0.20	−0.06	0.19	0.16	0.10	0.29	0.11
SJ (cm)	−0.03	−0.01	−0.07	−0.04	0.03	0.11	0.21	−0.06	0.21	0.35	−0.01	−0.04	0.07	−0.01	0.25
CMJ (cm)	0.07	0.13	0.04	−0.02	−0.02	0.03	0.11	0.01	0.20	0.24	0.06	0.00	0.01	0.07	0.24
10-m (s)	0.15	0.13	0.21	0.14	0.07	−0.11	−0.11	0.17	−0.13	−0.34	0.16	0.10	−0.17	0.07	−0.29
30-m (s)	0.17	0.13	0.26	0.19	0.07	−0.24	−0.40	0.26	−0.15	−0.53 *	0.25	0.14	−0.25	0.09	−0.27
COD (s)	0.02	0.11	0.09	−0.10	−0.25	−0.46	−0.57 *	0.02	−0.25	−0.59 *	0.05	−0.12	−0.50 *	−0.04	−0.17
P_max_ (W)	0.00	0.08	−0.07	−0.13	0.01	0.14	0.21	−0.12	0.11	0.22	−0.08	−0.08	0.09	0.06	0.19
P_min_ (W)	0.26	0.30	0.21	0.17	0.26	0.23	0.20	0.18	0.33	0.09	0.22	0.20	0.13	0.25	0.27
FI (%)	−0.22	−0.13	−0.27	−0.32	−0.20	0.00	0.13	−0.32	−0.12	0.22	−0.29	−0.27	0.02	−0.13	0.03
YYIR1 (m)	0.12	−0.09	0.20	0.30	0.28	0.15	0.05	0.19	0.20	0.05	0.19	0.22	0.14	0.10	0.19

ADD: adductor strength; ABD: abductor strength; SJ: squat jump; CMJD: countermovement jump; COD: change-of-direction; P_max_: maximum power at repeated-sprint test; P_min_: minimum power at repeated-sprint test; P_average_: average power at repeated-sprint test; FI: fatigue index at repeated-sprint test; YYIR1: intermittent recovery test level 1; HR_max_: maximal heart rate; VO2_max_: maximal oxygen uptake; NS: number of sprints; MSA: maximum speed achieved; zone 1 (Z1), zone 2 (Z2), zone 3 (Z3), zone 4 (Z4), zone 5 (Z5), and zone 6 (Z6); acceleration (ace1, ace2, ace3) and deceleration (des1, des2, des3); D: distance covered; *: significant at *p* < 0.05.

**Table 4:** Correlations between fitness status and match running (assessment 3 and matches 7 to 10).

Measure	D	Z1	Z2	Z3	Z4	Z5	Z6	ace1	ace2	ace3	Des1	Des2	Des3	MSA	NS
ADD (kg)	−0.02	−0.05	0.05	0.11	−0.07	−0.31	−0.50	0.07	−0.22	−0.22	0.12	−0.07	−0.37	−0.23	−0.15
ABD (kg)	−0.10	−0.13	−0.03	0.01	−0.15	−0.32	−0.41	−0.07	−0.28	−0.17	−0.03	−0.16	−0.28	−0.27	−0.19
SJ (cm)	0.46	0.48	0.34	0.45	0.53	0.57	0.56	0.44	0.59	0.76 *	0.42	0.64 *	0.63 *	0.46	0.70 *
CMJ (cm)	0.60	0.61 *	0.50	0.60	0.63 *	0.60	0.48	0.57	0.64 *	0.69 *	0.56	0.68 *	0.63 *	0.56	0.70 *
10-m (s)	−0.52	−0.51	−0.47	−0.52	−0.52	−0.47	−0.43	−0.52	−0.61 *	−0.55	−0.49	−0.60 *	−0.56	−0.55	−0.75 *
30-m (s)	−0.57	−0.59	−0.47	−0.48	−0.57	−0.63 *	−0.70 *	−0.53	−0.68 *	−0.77 *	−0.51	−0.68 *	−0.68 *	−0.67 *	−0.80 *
COD (s)	−0.49	−0.51	−0.29	−0.45	−0.68 *	−0.80 *	−0.77 *	−0.50	−0.76 *	−0.84 *	−0.45	−0.78 *	−0.75 *	−0.56	−0.74 *
P_max_ (W)	0.43	0.52	0.39	0.21	0.23	0.30	0.36	0.35	0.30	0.51	0.30	0.41	0.31	0.51	0.49
P_min_ (W)	0.12	0.17	0.11	−0.01	0.02	0.12	−0.01	0.08	0.02	0.16	0.05	0.14	0.07	0.04	0.35
FI (%)	0.47	0.56	0.43	0.27	0.28	0.31	0.46	0.39	0.36	0.54	0.35	0.43	0.34	0.62 *	0.42
YYIR1 (m)	−0.41	−0.47	−0.42	−0.28	−0.19	−0.08	−0.25	−0.36	−0.19	−0.30	−0.35	−0.27	−0.12	−0.56	−0.08

ADD: adductor strength; ABD: abductor strength; SJ: squat jump; CMJD: countermovement jump; COD: change-of-direction; P_max_: maximum power at repeated-sprint test; P_min_: minimum power at repeated-sprint test; P_average_: average power at repeated-sprint test; FI: fatigue index at repeated-sprint test; YYIR1: intermittent recovery test level 1; HR_max_: maximal heart rate; VO2_max_: maximal oxygen uptake; NS: number of sprints; MSA: maximum speed achieved; zone 1 (Z1), zone 2 (Z2), zone 3 (Z3), zone 4 (Z4), zone 5 (Z5), and zone 6 (Z6); acceleration (ace1, ace2, ace3) and deceleration (des1, des2, des3); D: distance covered; *: significant at *p* < 0.05.

**Table 5:** Values of regression analysis explaining fitness assessment and match running performance.

Measure	Measure	Assessment 2 and Matches 1–4					
		b *	SE of b *	R^2^	Adjusted R^2^	F	*p*
30-m (s)	ace3 (n)	−0.39	0.21	0.15	0.10	3.29	0.08
COD (s)	Z6 (m)	−0.55	0.19	0.30	0.25	7.71	0.01 *
	ace3 (n)	−0.56	0.19	0.31	0.27	8.36	0.009 *
	des3 (n)	−0.49	0.20	0.24	0.19	5.71	0.027 *
		**Assessment 3 and matches 7–10**					
SJ (cm)	ace3 (n)	0.50	0.22	0.25	0.20	5.04	0.04 *
	des2 (n)	0.27	0.24	0.77	0.01	1.25	0.28
	des3 (n)	0.36	0.24	0.13	0.07	2.25	0.15
	NS (n)	0.43	0.23	0.18	0.13	3.45	0.08
CMJ (cm)	Z1 (m)	0.33	0.24	0.11	0.05	1.93	0.18
	Z4 (m)	0.39	0.23	0.15	0.10	2.78	0.11
	ace2 (n)	0.35	0.24	0.12	0.07	2.14	0.16
	ace3 (n)	0.50	0.22	0.25	0.20	4.98	0.04 *
	des2 (n)	0.34	0.24	0.11	0.05	1.93	0.18
	des3 (n)	0.40	0.23	0.15	0.10	2.79	0.11
	NS (n)	0.45	0.23	0.45	0.15	3.95	0.06
10-m (s)	ace2 (n)	−0.19	0.24	0.37	-	0.61	0.44
	des2 (n)	−0.12	0.25	0.16	-	0.26	0.61
	NS (n)	−0.33	0.23	0.11	0.06	2.04	0.17
30-m (s)	Z5 (m)	−0.25	0.24	0.01	0.01	1.13	0.03
	Z6 (m)	−0.10	0.24	0.01	-	0.17	0.68
	Ace2 (n)	−0.26	0.24	0.07	0.01	1.18	0.29
	Ace3 (n)	−0.18	0.25	0.03	-	0.56	0.46
	Des2 (n)	−0.20	0.25	0.03	-	0.65	0.42
	Des3 (n)	−0.23	0.24	0.05	-	0.88	0.36
	MSA (km/h)	−0.39	0.23	0.12	0.09	3.11	0.19
	NS (n)	−0.41	0.22	0.16	0.11	3.24	0.09
COD (s)	Z4 (m)	−0.49	0.22	0.24	0.19	4.84	0.04 *
	Z5 (m)	−0.57	0.21	0.31	0.27	7.04	0.01 *
	Z6 (m)	−0.33	0.24	0.10	0.05	1.83	0.19
	Ace2 (n)	−0.55	0.22	0.30	0.25	6.53	0.02 *
	Ace3 (n)	−0.40	0.23	0.16	0.10	2.92	0.10
	Des2 (n)	−0.54	0.22	0.23	0.24	6.31	0.02 *
	Des3 (n)	−0.48	0.23	0.23	0.18	4.45	0.052
	NS (n)	−0.59	0.21	0.35	0.30	7.98	0.01 *
FI (%)	MSA (km/h)	−0.08	0.25	0.01	-	0.10	0.75

SJ: squat jump; CMJD: countermovement jump; COD: change-of-direction; P_max_: maximum power at repeated-sprint test; P_min_: minimum power at repeated-sprint test; P_average_: average power at repeated-sprint test; FI: fatigue index at repeated-sprint test; YYIR1: intermittent recovery test level 1; HR_max_: maximal heart rate; VO2_max_: maximal oxygen uptake; NS: number of sprints; MSA: maximum speed achieved; zone 1 (Z1), zone 2 (Z2), zone 3 (Z3), zone 4 (Z4), zone 5 (Z5) and zone 6 (Z6); acceleration (ace1, ace2, ace3) and deceleration (des1, des2, des3); D: distance covered; *: significant at *p* < 0.05.

## References

[1] Manson S., Brughelli M., Harris N. (2014). Physiological Characteristics of International Female Soccer Players. J. Stregth Cond. Res..

[2] Krustrup P., Mohr M., Ellinsgaard H., Bangsbo J. (2005). Physical Demands during an Elite Female Soccer Game: Importance of Training Status. Med. Sci. Sports Exerc..

[3] Milanović Z., Sporiš G., James N., Trajković N., Ignjatović A., Sarmento H., Trecroci A., Mendes B. (2017). Physiological Demands, Morphological Characteristics, Physical Abilities and Injuries of Female Soccer Players. J. Hum. Kinet..

[4] Davis J., Brewer J. (1993). Applied Physiology of Female Soccer Players. Sports Med..

[5] Stepinski M., Ceylan H.I., Zwierko T. (2020). Seasonal Variation of Speed, Agility and Power Performance in Elite Female Soccer Players: Effect of Functional Fitness. Phys. Activ. Rev..

[6] Datson N., Hulton A., Andersson H., Lewis T., Weston M., Drust B., Gregson W. (2015). Applied Physiology of Female Soccer: An Update. Sports Med..

[7] Datson N., Drust B., Weston M., Jarman I., Lisboa P., Gregson W. (2017). Match Physical Performance of Elite Female Soccer Player during International Competition. J. Stregth Cond. Res..

[8] Trewin J., Meylan C., Varley M.C., Cronin J. (2018). The Match-to-Match Variation of Match-Running in Elite Female Soccer. J. Sci. Med. Sport.

[9] Vescovi J.D. (2012). Sprint Profile of Professional Female Soccer Players during Competitive Matches: Female Athletes in Motion (FAiM) study. J. Sports Sci..

[10] Haugen T., Tønnessen E., Seiler S. (2012). Speed and Countermovement-Jump Characteristics of Elite Female Soccer Players, 1995–2010. Int. J. Sports Physiol. Perform..

[11] McCormack W., Stout J., Wells A., Gonzalez A., Mangine G., Hoffman J. (2014). Predictors of High-Intensity Running Capacity in Collegiate Women during a Soccer Game. J. Stregth Cond. Res..

[12] Andersson H., Randers M., Heiner-Moller A., Krustrup P., Mohr M. (2010). Elite Female Soccer Players Perform More High-Intensity Running When Playing in International Games Compared with Domestic League Games. J. Stregth Cond. Res..

[13] Niessen M., Hartmann U., Martı V. (2014). ScienceDirect Women’s Football: Player Characteristics and Demands of the Game. J. Sport Health Sci..

[14] Hoare D.G., Warr C.R. (2010). Talent Identification and Women’s Soccer: An Australian Experience. J. Sport Sci..

[15] Andersson H., Raastad T., Nilsson J., Paulsen G.K.R.A.N., Garthe I.N.A., Kadi F. (2008). Neuromuscular Fatigue and Recovery in Elite Female Soccer: Effects of Active Recovery. Med. Sci. Sports Exerc..

[16] Sjökvist J., Laurent M., Richardson M., Curtner-Smith M., Holmber H.-C., Bishop P. (2014). Recovery from High-intensity Training Sessions in Female Soccer Players. J. Stregth Cond. Res..

[17] Paulsen K., Butts C., McDermott B. (2018). Observation of women soccer players’ physiology during a single season. J. Stregth Cond. Res..

[18] Castagna C., Castellini E. (2013). Vertical Jump Performance in Italian Male and Female National Team Soccer Players. J. Strength Cond. Res..

[19] Sedano S., Vaeyens R., Philippaerts R., Redondo J., Cuadrado G. (2009). Anthropometric and Anerobic Fitness Profile of Elite and Non-Elite Female Soccer Players. J. Sports Med. Phys. Fit..

[20] Krustrup P., Zebis M., Jensen J.M., Mohr M. (2010). Game-Induced Fatigue Patterns in Elite Female Soccer. J. Strength Cond. Res..

[21] Mujika I., Santisteban J., Impellizzeri F.M., Castagna C. (2009). Fitness Determinants of Success in Men’s and Women’s Football. J. Sport Sci..

[22] Mara J., Thompson K., Pumpa K.L., Ball N. (2015). Periodisation and Physical Performance in Elite Female Soccer Players. Int. J. Sports Physiol. Perform..

[23] Rampinini E., Coutts A.J., Castagna C., Sassi R., Impellizzeri F.M. (2007). Variation in Top Level Soccer Match Performance. Int. J. Sports Med..

[24] Buchheit M., Mendez-Villanueva A., Quod M.J., Poulos N., Bourdon P. (2010). Determinants of the Variability of Heart Rate Measures during a Competitive Period in Young Soccer Players. Eur. J. Appl. Physiol..

[25] Haddad H., Simpson B.M., Buchheit M., Di Salvo V., Mendez-Villanueva A. (2015). Peak Match Speed and Maximal Sprinting Speed in Young Soccer Players: Effect of Age and Playing Position. Int. J. Sports Physiol. Perform..

[26] Buchheit M., Saint P., Football G., Mendez-villanueva A., Simpson B.M., Bourdon P.C. (2010). Match Running Performance and Fitness in Youth Soccer. Int. J. Sports Med..

[27] Aquino R., Carling C., Maia J., Vieira L.H.P., Wilson R.S., Smith N., Almeida R., Gonçalves L.G.C., Kalva-Filho C.A., Garganta J. (2020). Relationships between Running Demands in Soccer Match-Play, Anthropometric, and Physical Fitness Characteristics: A Systematic Review. Int. J. Perform. Anal. Sport.

[28] Stolen T., Chamari K., Castagna C., Wisloff U. (2005). Physiology of Soccer. Sports Med..

[29] Castellano J., Alvarez-Pastor D., Bradley P.S. (2014). Evaluation of Research Using Computerised Tracking Systems (Amisco and Prozone) to Analyse Physical Performance in Elite Soccer: A Systematic Review. Sports Med. Auckl. N. Z..

[30] Carling C., Nelson L. (2008). The Role of Motion Analysis in Elite Soccer: Contemporary Performance Measurements Techniques and Work Rate Data. Sports Med..

[31] Carling C. (2013). Interpreting Physical Performance in Professional Soccer Match-Play: Should We Be More Pragmatic in Our Approach?. Sports Med. Auckl. N. Z..

[32] Bangsbo J., Mohr M., Krustrup P. (2006). Physical and Metabolic Demands of Training and Match-Play in the Elite Foot- Ball Player. J. Sports Sci..

[33] Castagna C., Impellizzeri F.M., Manzi V., Ditroilo M. (2010). The Assessment of Maximal Aerobic Power with the Multistage Fitness Test in Young Women Soccer Players. J. Strength Cond. Res..

[34] Krustrup P., Mohr M., Amstrup T., Rysgaard T., Johansen J., Steensberg A., Pedersen P.K., Bangsbo J. (2003). The Yo-Yo Intermittent Recovery Test: Physiological Response, Reliability, and Validity. Med. Sci. Sports Exerc..

[35] Fernandes-da-Silva J., Castagna C., Teixeira A.S., Carminatti L.J., Guilherme L., Guglielmo A. (2016). The Peak Velocity Derived from the Carminatti Test Is Related to Physical Match Performance in Young Soccer Players The Peak Velocity Derived from the Carminatti Test Is Related to Physical Match. J. Sports Sci..

[36] Aquino R., Vieira L.H.P., Oliveira L.D.P., Gonçalves L.G.C., Santiago P.R.P. (2018). Relationship between Field Tests and Match Running Performance in High-Level Young Brazilian Soccer Players. J. Sports Med. Phys. Fit..

[37] Castagna C., Impellizzeri F., Cecchini E.M.C., Rampinini E., Alvarez J. (2009). Effects of Intermittent-Endurance Fitness on Match Performance in Young Male Soccer Players. J. Stregth Cond. Res..

[38] Castagna C., Manzi V., Impellizzeri F., Weston M., Barbero Alvarez J.C. (2010). Relationship Between Endurance Field Tests and Match Performance in Young Soccer Players. J. Strength Cond. Res..

[39] Rago V., Silva J.R., Mohr M., Barreira D., Krustrup P., Rago V., Silva J.R., Mohr M., Barreira D., Krustrup P. (2018). The Inter-Individual Relationship between Training Status and Activity Pattern during Small-Sided and Full-Sized Games in Professional Male Football Players. Sci. Med. Footb..

[40] Rebelo A., Brito J., Seabra A., Oliveira J., Krustrup P. (2014). Physical Match Performance of Youth Football Players in Relation to Physical Capacity. Eur. J. Sport Sci..

[41] Abt G., Lovell R.I.C. (2009). The Use of Individualized Speed and Intensity Thresholds for Determining the Distance Run at High-Intensity in Professional Soccer. J. Sports Sci..

[42] Mohr M., Krustrup P., Bangsbo J. (2003). Match Performance of High-Standard Soccer Players with Special Reference to Development of Fatigue. J. Sports Sci..

[43] Cipryan L., Gajda V. (2011). The Influence of Aerobic Power on Repeated Anaerobic Exercise in Junior Soccer Players. J. Hum. Kinet..

[44] Granier P., Mercier B., Mercier J., Anselme F., Préfaut C. (1995). Aerobic and Anaerobic Contribution to Wingate Test Performance in Sprint and Middle-Distance Runners. Eur. J. Appl. Physiol..

[45] Metaxas T.I. (2021). Match Running Performance of Elite Soccer Players: Vo2max and Players Position Influences. J. Strength Cond. Res..

[46] Redkva P.E., Paes M.R., Fernandez R., da-Silva S.G. (2018). Correlation between Match Performance and Field Tests in Professional Soccer Players. J. Hum. Kinet..

[47] Aslan A., Acikada C., Güvenç A., Gören H., Hazir T., Özkara A. (2012). Metabolic Demands of Match Performance in Young Soccer Players. J. Sports Sci. Med..

[48] Carling C., Le Gall F., McCall A., Nedelec M., Dupont G. (2013). Are Aerobic Fitness and Repeated Sprint Ability Linked to Fatigue in Professional Soccer Match-Play? A Pilot Study. J. Athl. Enhanc..

[49] Bangsbo J., Lindquist F. (1992). Comparison of Various Exercise Tests with Endurance Performance during Soccer in Professional Players. Int. J. Sports Med..

[50] Palucci Vieira L.H., Arins F.B., Guglielmo L.G.A., de Lucas R.D., Carminatti L.J., Santiago P.R.P. (2021). Game Running Performance and Fitness in Women’s Futsal. Int. J. Sports Med..

[51] Mendez-Villanueva A., Buchheit M. (2011). Physical Capacity–Match Physical Performance Relationships in Soccer: Simply, More Complex. Eur. J. Appl. Physiol..

[52] Paul D.J., Nassis G.P. (2015). Physical Fitness Testing in Youth Soccer: Issues and Considerations Regarding Reliability, Validity and Sensitivity. Pediatr. Exerc. Sci..

[53] Bradley P.S., Mohr M., Bendiksen M., Randers M.B., Flindt M., Barnes C., Hood P., Gomez A., Andersen J.L., Di Mascio M. (2011). Sub-Maximal and Maximal Yo–Yo Intermittent Endurance Test Level 2: Heart Rate Response, Reproducibility and Application to Elite Soccer. Eur. J. Appl. Physiol..

[54] Aquino R., Melli-Neto B., Ferrari J.V.S., Bedo B.L.S., Vieira L.H.P., Santiago P.R.P., Gonçalves L.G.C., Oliveira L.P., Puggina E.F. (2019). Validity and Reliability of a 6-a-Side Small-Sided Game as an Indicator of Match-Related Physical Performance in Elite Youth Brazilian Soccer Players. J. Sports Sci..

[55] Stevens T.G.A., Ruiter C.J.D., Beek P.J., Savelsbergh G.J.P. (2016). Validity and Reliability of 6-a-Side Small-Sided Game Locomotor Performance in Assessing Physical Fitness in Football Players. J. Sports Sci..

[56] Aquino R., Munhoz Martins G.H., Palucci Vieira L.H., Menezes R.P. (2017). Influence of Match Location, Quality of Opponents, and Match Status on Movement Patterns in Brazilian Professional Football Players. J. Strength Cond. Res..

[57] Granero-Gil P., Bastida-Castillo A., Rojas-Valverde D., Gómez-Carmona C.D., de la Cruz Sánchez E., Pino-Ortega J. (2020). Influence of Contextual Variables in the Changes of Direction and Centripetal Force Generated during an Elite-Level Soccer Team Season. Int. J. Environ. Res. Public Health.

[58] Fitzpatrick J.F., Linsley A., Musham C. (2019). Running the Curve: A Preliminary Investigation into Curved Sprinting during Football Match-Play. Sport Perform. Sci. Rep..

[59] Schimpchen J., Skorski S., Nopp S., Meyer T. (2016). Are “Classical” Tests of Repeated-Sprint Ability in Football Externally Valid? A New Approach to Determine in-Game Sprinting Behaviour in Elite Football Players. J. Sports Sci..

[60] Buchheit M., Simpson B.M., Hader K., Lacome M. (2020). Occurrences of Near-to-Maximal Speed-Running Bouts in Elite Soccer: Insights for Training Prescription and Injury Mitigation. Sci. Med. Footb..

[61] Prendergast N., Hopper D., Finucane M., Grisbrook T.L. (2016). Hip Adduction and Abduction Strength Profiles in Elite, Sub-Elite and Amateur Australian Footballers. J. Sci. Med. Sport.

[62] Hannigan J.J., Osternig L.R., Chou L.-S. (2018). Sex-Specific Relationships Between Hip Strength and Hip, Pelvis, and Trunk Kinematics in Healthy Runners. J. Appl. Biomech..

[63] Brund R.B.K., Rasmussen S., Nielsen R.O., Kersting U.G., Laessoe U., Voigt M. (2018). The Association between Eccentric Hip Abduction Strength and Hip and Knee Angular Movements in Recreational Male Runners: An Explorative Study. Scand. J. Med. Sci. Sports.

[64] Pino-Ortega J., Rojas-Valverde D., Gómez-Carmona C.D., Rico-González M. (2021). Training Design, Performance Analysis, and Talent Identification—A Systematic Review about the Most Relevant Variables through the Principal Component Analysis in Soccer, Basketball, and Rugby. Int. J. Environ. Res. Public Health.

[65] Harper D.J., Carling C., Kiely J. (2019). High-Intensity Acceleration and Deceleration Demands in Elite Team Sports Competitive Match Play: A Systematic Review and Meta-Analysis of Observational Studies. Sports Med. Auckl. N. Z..

[66] Mara J.K., Thompson K.G., Pumpa K.L., Morgan S. (2017). The Acceleration and Deceleration Profiles of Elite Female Soccer Players during Competitive Matches. J. Sci. Med. Sport.

[67] Gregson W., Drust B., Atkinson G., Salvo V.D. (2010). Match-to-Match Variability of High-Speed Activities in Premier League Soccer. Int. J. Sports Med..

